# Laboratory Screening of Control Agents Against Isolated Fungal Pathogens Causing Postharvest Diseases of Pitaya in Guizhou, China

**DOI:** 10.3389/fchem.2022.942185

**Published:** 2022-06-30

**Authors:** Yong Li, Haijiang Chen, Lan Ma, Youshan An, Hui Wang, Wenneng Wu

**Affiliations:** Food and Pharmaceutical Engineering Institute, Guiyang University, Guiyang, China

**Keywords:** pitaya, postharvest disease, pathogen identification, drug sensitivity test, plant extracts

## Abstract

Pitaya, or dragon fruit, is a typical tropical fruit with an appealing taste and diverse health benefits to humans. The plantation of pitaya in Guizhou province in China has greatly boosted the income of local farmers and alleviated poverty. However, the frequent occurrence of postharvest diseases has brought large economic loss. To find a solution, we set out to identify the postharvest disease-causing agents of Guizhou pitaya. Several fungi were isolated from diseased pitaya and identified as species based on the ITS1 sequence similarity. Of them, *Penicillium spinulosum*, *Phoma herbarum*, *Nemania bipapillata*, and *Aspergillus oryzae* were, for the first time, found to cause dragon fruit disease. In consideration of their prevalence in postharvest fruit diseases, *Alternaria alternata* H8 and *Fusarium proliferatum* H4 were chosen as representative pathogens for the drug susceptibility test. Among the tested drugs and plant extracts, 430 g/L tebuconazole and 45% prochloraz were found to be the most potent fungicides against H8 and H4, respectively. The research provides insights into the mechanism and control of postharvest diseases of dragon fruits in Guizhou, China, and thus could be of economic and social significance to local farmers and the government.

## Introduction

Dragon fruit or pitaya (*Hylocereus species*) belongs to the family Cataceae, which is a typical tropical fruit. Since its introduction to Guizhou province in 2001 (Wang et al., 2018), the fruit has been shown to be quite adapted to the local climate and ecology. So far, three varieties, purple dragon, crystal red dragon, and pink dragon, have been introduced and cultivated in Guizhou, and the dragon fruit-planting area in Guizhou province has increased to be the third in China (Wang et al., 2018). With its renowned health benefits to consumers and appealing taste, the locally produced dragon fruit is widely accepted in the domestic market. Dragon fruit cultivation in Guizhou province has greatly boosted the local economy and been lifting local farmers out of poverty. With the planting history and area growing, the incidences of dragon fruit diseases are increasingly more frequent, especially the postharvest diseases, which lead to the decline of the yield and quality of marketable dragon fruits, affecting the economic benefits ultimately.

Due to the interruption of nutrient supply, the vitality of the postharvest dragon fruits was weakened, the disease resistance was reduced, and they could be infected easily by pathogenic microorganisms during storage and transportation, resulting in illness. At present, there are kinds of diseases caused by microorganisms such as anthracnose, soft rot, canker, black spot, and wilts in dragon fruit after harvest (Balendres and Bengoa, 2019). Among them, fungal diseases are more common and serious. Dragon fruit anthracnose was usually caused by *Colletotrichum gloeosporioides* (Masyahit et al., 2009) and *C. truncatum* (Guo et al., 2014). The pathogens that cause black spot disease of dragon fruit were reported to be *Alternaria alternata* (Castro et al., 2017) and *Bipolaris cactivora* (Tarnowski et al., 2010; Ben-Ze Ev et al., 2011). Dragon fruit soft rot is found to be caused by *Neoscytalidium dimidiatum* (Pan et al., 2021) and *Gilbertella persicaria* (Guo et al., 2012). However, there are few reports focusing on causative agents and control methods for postharvest diseases of dragon fruits in the Guizhou area.

In order to investigate the microbial species causing postharvest diseases on dragon fruits in Guizhou, samples of diseased dragon fruits were collected from the Luodian County of Guizhou province in China. Microbes were isolated from diseased fruit tissue and reinoculated on healthy fruits to confirm pathogenicity according to Koch’s rule. Pathogenic microorganisms were identified by rDNA-ITS sequence similarity analysis. Finally, a variety of prevention and control reagents were screened for inhibition efficacy against selected pathogens by an indoor experiment. This study provides a helpful understanding of the mechanism of postharvest diseases and control measures for dragon fruits in Guizhou province and the neighboring area.

## Materials and Methods

### Sample Collection and Microbial Isolation

Samples of diseased dragon fruits were collected in Luodian County in Guizhou Province. Pathogenic microorganisms were isolated by conventional tissue isolation methods in the laboratory. The potato dextrose agar (PDA) plates were used for separation and purification. The isolated strains were stored at −20°C in 40% glycerol.

### Pathogenicity Test

Healthy dragon fruits were selected and soaked in 75% alcohol for 1 min, followed by repeated washes in sterile water, and surface-dried. Mycelia “cakes” (blank agar “cakes” as control) were chopped aseptically from the culture plate and placed onto the surface (non-injury inoculation) and the stabbing wound (stab inoculation) of dragon fruits, which was then allowed to incubate at 28°C, and the status of infection was observed along during incubation.

### Morphological Characterization of Pathogens

The pathogenic microorganisms were cultured on PDA and incubated at 28°C. The colony morphology was checked regularly, and the mycelia were observed under a Model EX30 inverted microscope (Ningbo Shunyu Tech. Co. Ltd., Zhejiang, China).

### DNA Extraction and Molecular Identification

The fungus was cultured in potato dextrose broth (PDB) at 28°C for 3 days, and the mycelia were collected for genomic DNA (gDNA) extraction. Fungal gDNA was extracted according to the users’ instruction of the fungal genomic DNA rapid extraction kit (Sangon). The rDNA-ITS1 fragment of the gDNA was PCR-amplified using universal ITS1 primers and subjected to nucleotide sequencing. All the obtained sequences were searched for similarity against the NCBI nucleotide collection (nr) database with default parameters (https://blast.ncbi.nlm.nih.gov/Blast.cgi?PROGRAM=blastn&PAGE_TYPE=BlastSearch&LINK_LOC=blasthome). The phylogenetic tree was constructed using the neighbor-joining method to determine the taxonomic status.

### Preparation of Tested Fungicides and Plant Extracts

All tested fungicides are commercially available. The plant extracts were prepared as such: the plant was air-dried and milled to powder. For the procedure, 10 g of the powder was extracted with 95% ethanol and heated for 4 h with refluxing. The extract was filtered and evaporated under reduced pressure. The residue was re-dissolved with hot water, cooled, and adjusted to a concentration of 500 mg/mL as stock solution.

### Indoor Screening for Control Agents

Tested fungicide or plant extracts (Tween 20 as control) with appropriate quantity was added to melted PDA and cooled to make plates. The plates were then inoculated by placing an inoculum (4 mm disc from cultures of *A. alternata* and *F. proliferatum*) at the center and incubated at the ambient temperature of 28°C. Each test was performed in triplicate. After 6 days of incubation, the colony diameter was recorded, and the inhibition rate was calculated using the following formula:

$$\text{Inhibition}\;\text{rate}=\frac{diameter\;of\;control-diameter\;of\;treatment}{diameter\;of\;control-4\;mm}\times 100\%.$$

Furthermore, the efficacy for the tested control agent was expressed as the half-maximal effective concentration (EC_50_, the concentration at which the tested fungicide reduced mycelial growth by 50%) determined by regression of the inhibition rate against the log10 values of the fungicide concentrations.

## Results

### Isolation and Purification of Pathogenic Fungi

**FIGURE 1:**
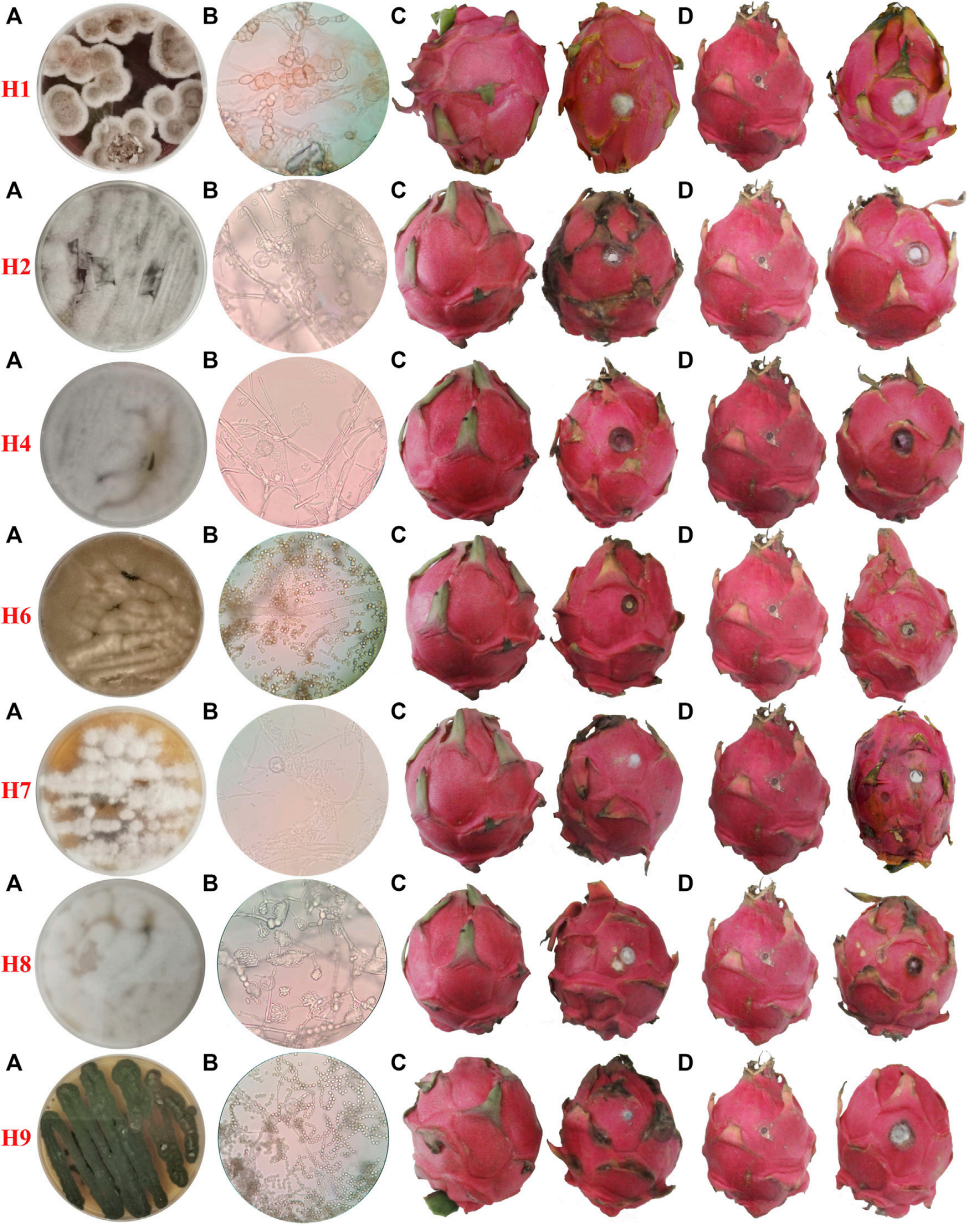
Morphological characteristics of pathogens and pathogenicity confirmation. **(A)** Colony morphology on the PDA plate. **(B)** Microscopic image of conidia taken with ×400 magnification, **(C)** non-injury inoculation, and **(D)** injury inoculation; fruits on the right were inoculated with mycelia “cakes,” and the ones on the left are blank controls.

A number of fungal isolates were separated from diseased dragon fruit tissue, and seven fungal strains were preliminarily established based on colony and mycelium morphology and labeled as H1, H2, H4, H6, H7, H8, and H9. Colony and conidia morphology are shown in Figure 1. The appearance of the H1 colony on PDA is brown with white slowly growing edges. The spores are elliptical to round with varying sizes but connected in tandem to each other. The H2 colony is whitish gray and grows fast. Its spores are rod-shaped under the microscope and distributed around the mycelia. The H4 strain colony exhibits a white appearance and grows fast with elliptical spores arranged in clusters around the top of the spore stalk. The colony of strain H6 is yellowish-brown with dense hyphae, and the spores appear round when observed under a microscope. The H7 colony is white in appearance and grows slowly on PDA. The spores are irregularly rod-shaped. The H8 colony is white with dense mycelia. The spores are oval under the microscope and densely clustered around spore stalks. The H9 colony is dark green and grows fast; the spores are spherical and connected in tandem to each other to form long chains.

### Pathogenicity of Isolated Microorganisms

The isolated strains were re-inoculated onto fruits in two ways, a non-injured inoculation and a stab-injured inoculation. As shown in Figure 1, all the seven strains can infect dragon fruits with or without an injury. Despite the presence of a stabbing wound, no observable infection occurred in control experiments where blank agar was used for ‘inoculation’.

### Taxonomic Identification

**FIGURE 2:**
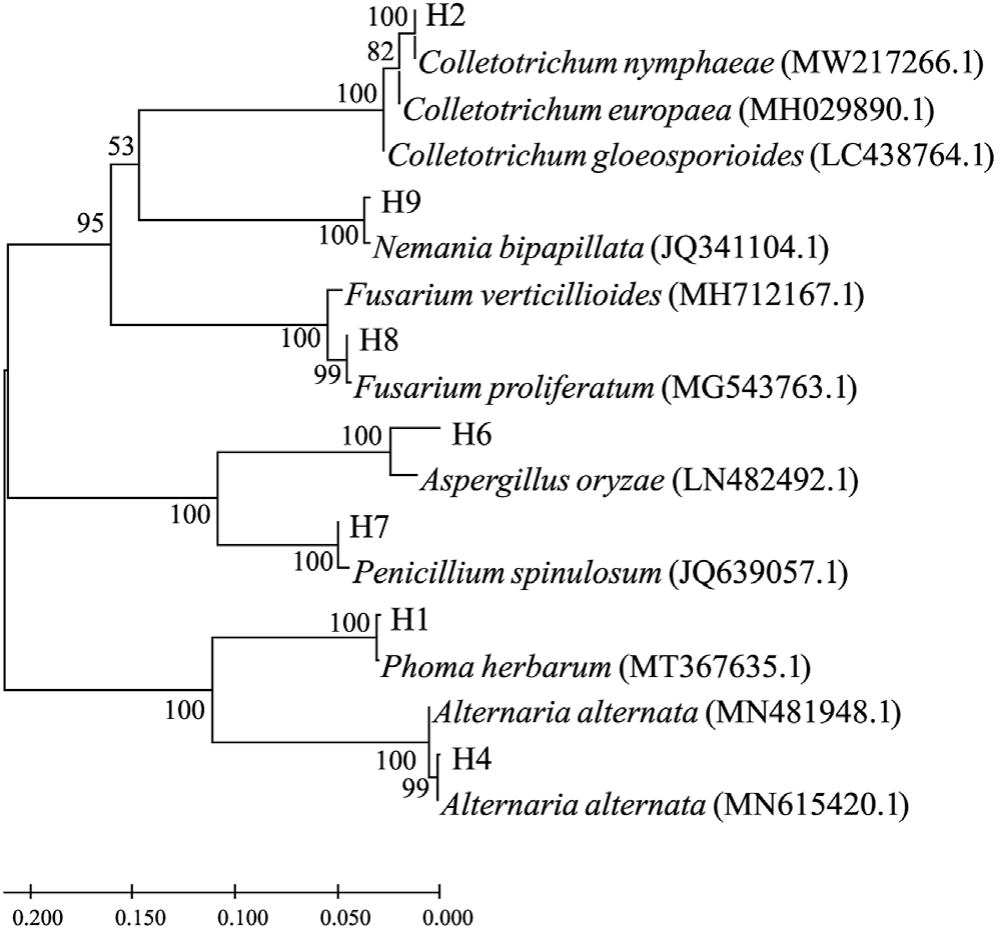
Molecular phylogenetic tree based on the rDNA-ITS sequence similarity. Sequence alignment and tree building were performed by MEGA5.0 using the neighbor-joining method, and phylogeny was tested by 500 bootstrap replications. The numbers on branches were calculated as bootstrap values. Accession numbers of ITS1 sequences from isolated strains were, namely, ON514545.1 (H1), ON514546.1 (H2), ON514547.1 (H4), ON514548.1 (H6), ON514549.1 (H7), ON514550.1 (H8), and ON514551.1 (H9).

The ITS1 segments were PCR-amplified from corresponding genomic DNA extracted from the seven pathogenic fungi. Their nucleotide sequences were used as a query for blast searches, and top hits are listed in Table 1. According to the search results, the pathogenic strains were roughly assigned as *Phoma herbarum* H1, *Colletotrichum nymphaeae* H2, *Alternaria alternata* H4, *Aspergillus oryzae* H6, *Penicillium spinulosum* H7, *Fusarium proliferatum* H8, and *Nemania bipapillata* H9. Phylogenetic relationships based on the ITS1 sequence similarity between the strains and selected top hits are illustrated in Figure 2.

**TABLE 1:** NCBI blast results of samples.

Strains	Taxonomy	Sequence similarity (%)	Top hit
H1	*Phoma herbarum*	99	MT367635.1
H2	*Colletotrichum nymphaeae*	100	MW217266.1
H4	*Alternaria alternata*	100	MN944587.1
H6	*Aspergillus oryzae*	99	MH345908.1
H7	*Penicillium spinulosum*	100	JQ639057.1
H8	*Fusarium proliferatum*	99	MG543763.1
H9	*Nemania bipapillata*	99	JQ341104.1

### Preliminary Screening of Agro-Agents for Fungicides Against Representative Pathogenic Fungi

In consideration of the prevalence of *A. alternata* and *F. proliferatum* in fruit disease, the two strains H4 and H8 were chosen as representative pathogenic microorganisms of dragon fruit and tested their susceptibility to a series of agricultural agents. As shown in Table 2, a total of 20 agro-agents were screened for indoor toxicity at concentrations of 50 μg/ml and 10 μg/ml on *A. alternata* H4 and *F. proliferatum* H8. For *A. alternata* H4, at 50 μg/ml concentration, 10% difenoconazole, 430 g/L tebuconazole, and 3% zhongshengmycin showed the highest inhibition rate, while at 10 *μ*g/ml concentration, 10% difenoconazole, 430 g/L tebuconazole, 50% iprodione were the three most potent candidates. For *F. proliferatum* H8, 3% benziothiazolinone, 430 g/L tebuconazole, and 45% prochloraz exhibited the highest toxicity at a concentration of 50 μg/ml, while at the 10 μg/ml level, 10% difenoconazole, 50% iprodione, and 45% prochloraz were the top three candidates. Notably, three microbial preparations were included in the screening; the insecticidal *Beauveria bassiana* showed the highest inhibition rate at 50 μg/ml concentration, and the fungicidal *Bacillus cereus* was the most potent at 10 μg/ml.

**TABLE 2:** Inhibitory effects of 20 agro-agents at the concentrations of 50 µg/ml and 10 µg/ml on *A. alternata* H4 and *F. proliferatum* H8.

Agro-agents	Inhibition rate (%)			
	50 µg/ml		10 µg/ml	
	*A. alternata* H4	*F. proliferatum* H8	*A. alternata* H4	*F. proliferatum* H8
80% mancozeb	47.39 ± 1.72	33.52 ± 2.34	6.43 ± 1.93	17.96 ± 1.51
10% difenoconazole	92.46 ± 2.85	87.92 ± 3.60	80.76 ± 2.93	82.93 ± 3.37
2% wuyiencin	52.14 ± 2.40	56.36 ± 2.58	46.71 ± 2.91	38.08 ± 2.93
3% benziothiazolinone	73.68 ± 2.78	84.05 ± 3.56	54.44 ± 2.80	46.54 ± 1.38
50% chloroisobromine cyanuric acid	10.61 ± 2.33	12.49 ± 2.56	4.96 ± 2.80	6.01 ± 1.69
50% iprodione	70.25 ± 1.63	74.32 ± 2.79	56.37 ± 3.67	67.54 ± 2.79
50% benzylpenicillin	39.78 ± 1.61	56.12 ± 2.78	5.26 ± 2.70	16.51 ± 1.75
80% ethylicin	76.09 ± 2.85	82.54 ± 2.24	54.11 ± 2.00	61.54 ± 2.41
3% zhongshengmycin	82.90 ± 3.64	75.74 ± 2.54	29.91 ± 1.20	27.04 ± 2.01
75% chlorothalonil	54.32 ± 3.01	68.67 ± 1.89	48.44 ± 2.89	49.20 ± 2.07
50% sulfur·carbendazim	30.25 ± 1.46	19.27 ± 1.04	7.93 ± 2.44	3.58 ± 2.19
20% bismerthiazol	42.98 ± 1.42	52.10 ± 1.27	24.29 ± 2.21	35.12 ± 2.22
72% agricultural streptomycin sulfate	25.62 ± 2.81	18.96 ± 2.33	11.84 ± 2.77	8.49 ± 2.86
70% thiophanate methyl	13.10 ± 1.16	32.64 ± 2.64	5.35 ± 1.02	5.21 ± 2.57
50% kresoxim methyl	82.47 ± 1.92	68.79 ± 2.63	47.47 ± 1.39	64.79 ± 2.63
430 g/L tebuconazole	94.26 ± 301	95.46 ± 1.18	84.72 ± 3.61	75.46 ± 1.73
45% prochloraz	63.25 ± 2.51	98.79 ± 1.73	10.26 ± 1.85	84.26 ± 1.42
*Bacillus cereus* preparation (8*10^9^ spores/g)	48.00 ± 2.06	42.61 ± 2.94	17.19 ± 2.83	20.69 ± 1.84
*Beauveria bassiana* preparation (2*10^11^ spores/g)	51.76 ± 2.99	78.96 ± 3.01	6.14 ± 2.28	3.73 ± 2.39
*Verticillium chlamydosporium* preparation (2.5 * 10^9^ spores/g)	28.82 ± 1.70	17.13 ± 2.12	6.14 ± 2.64	12.25 ± 2.60

### Inhibition Effect of 10 Edible and Medicinal Plant Extracts on the Tested Fungus

The ethanol extract was obtained from 10 edible and medicinal plants, namely, *Houttuynia cordata* Thunb, *Mentha haplocalyx*, *Zanthoxylum bungeanum* Maxim, *Lonicera japonica* Thunb, *Dendrobium officinale* Kimura et Migo, *Piper nigrum, Zingiber officinale* Roscoe, *Gastrodia elata*, *Schisandra chinensis*, and *Illicium verum*. Some of the plants are renowned for their microbe-inhibitory activity. The inhibition rate results in this study are shown in Table 3. All 10 ethanolic extracts prepared at a final concentration of 50 mg/ml showed an inhibitory effect on the two fungal pathogens, *A. alternata* H4 and *F. proliferatum* H8. In comparison, 50 mg/ml and 80% ethylicin was included in the test. The inhibition rates of *Zanthoxylum bungeanum* Maxim against *A. alternata* H4 and *F. proliferatum* H8 were 68.75 and 75.12%, those of *Zingiber officinale* Roscoe were 70.14 and 60.48%, and those of *Piper nigrum* were 69.84 and 60.93%, respectively. The three extracts showed the highest inhibitory effect against both tested pathogens of dragon fruit. Notably, 80% ethylicin at the same concentration exhibited a 100% inhibition rate in both strains.

**TABLE 3:** Determination of the inhibitory effect of 10 Chinese edible and medicinal plant extracts on A. *alternata* H4 and *F. proliferatum* H8.

Plant extract	Inhibition rate (%)		Plant extract	Inhibition rate (%)	
	*A. alternata* H4	*F. proliferatum* H8		*A. alternata* H4	*F. proliferatum* H8
*Zanthoxylum bungeanum *Maxim	68.75 ± 1.15	75.12 ± 2.38	*Mentha haplocalyx*	7.24 ± 1.03	32.48 ± 1.54
*Lonicera japonica* Thunb	11.27 ± 1.72	34.84 ± 1.69	*Dendrobium officinale* Kimura et Migo	37.94 ± 1.35	26.75 ± 1.82
*Zingiber officinale* Roscoe	70.14 ± 2.16	60.48 ± 1.37	*Piper nigrum*	69.84 ± 1.19	60.93 ± 1.79
*Illicium verum*	37.68 ± 1.27	52.48 ± 1.77	*Schisandra chinensis*	48.37 ± 2.42	35.17 ± 2.80
*Houttuynia cordata* Thunb	24.64 ± 1.93	45.11 ± 2.60	*Gastrodia elata*	15.24 ± 1.91	21.68 ± 2.54
80% ethylicin	100	100			

### Fungicidal Efficacy Test on Representative Fungi With Promising Candidates

Based on previousscreening results, several fungicides were selected for the efficacy test. As shown in Table 4, 430 g/L tebuconazole exhibited the smallest EC_50_ at 0.0133 μg/ml, suggesting the highest potency toward *A. alternata* H4, while 50% iprodione with EC_50_ at 3.6840 μg/ml showed the second highest potency. For the pathogen *F. proliferatum* H8, 45% prochloraz showed the smallest EC_50_ at 0.0122 μg/ml, and 430 g/L tebuconazole is the second smallest with EC_50_ at 0.0307 μg/ml. It is worth mentioning that the efficacy of the biological agent *Bacillus cereus* is also tested, which exhibited EC_50_ at 81.3915 μg/ml toward *F. proliferatum* H8.

**TABLE 4:** Determination of the efficacy of screened fungicides to *A. alternata* H4 and *F. proliferatum* H8.

Pathogenic strain	Control agent	Regression equation	Correlation coefficient	EC50 (µg/ml)
*A. alternata* H4	3% benziothiazolinone	y = 0.8324x+4.1566	0.9770	10.3089
	10% difenoconazole	y = 0.7454x+4.2309	0.9871	10.7595
	50% iprodione	y = 0.8428x+4.5227	0.9934	3.6840
	50% kresoxim-methyl	y = 0.5347x+4.3594	0.9813	15.7781
	80% ethylicin	y = 1.0551x+3.4653	0.9956	28.4809
	430 g/L tebuconazole	y = 0.2501x+5.4687	0.9254	0.0133
*F. proliferatum* H8	10% difenoconazole	y = 0.3704x+5.1064	0.9903	0.5161
	45% prochloraz	y = 0.8479x+6.6203	0.9013	0.0122
	50% iprodione	y = 0.9036x+4.2205	0.9953	7.2888
	50% kresoxim-methyl	y = 0.3352x+4.7836	0.9766	4.4216
	80% ethylicin	y = 0.9706x+3.8647	0.9959	14.7804
	430 g/L tebuconazole	y = 0.7926x+6.1988	0.9111	0.0307

## Discussion

This study descripted the isolation and identification of several pathogenic fungal strains from diseased dragon fruits suffering from soft rot, anthracnose, and black spot based on field observation. The capability of the pathogens to infect healthy dragon fruits was confirmed by re-inoculation. Based on the morphological and molecular characteristics, the isolates were roughly identified to be *P. herbarum* H1, *C. nymphaeae* H2, *A. alternata* H4, *A. oryzae* H6, *P. spinulosum* H7, *F. proliferatum* H8, and *N. bipapillata* H9. Previous studies have suggested that a variety of *Fusarium* can cause postharvest soft rot disease to dragon fruit (Oeurn et al., 2015), but few studies report on *F. proliferatum*, which is found for the first time in the study in Guizhou province, suggesting that *F. proliferatum* could be a regional pathogen. *C. gloeosporioides* (Bordoh et al., 2020) and *C. truncatum* (Guo et al., 2014; Vijaya et al., 2015) of *Colletotrichum* genera were recognized as the pathogenic microorganisms of pitaya anthracnose. *C. nymphaeae* was reported to cause anthrax in other fruits such as plum (Chang et al., 2018) and blueberry (Tomoo et al., 2015), and this is the first time found in diseased pitaya. *A. alternata* is the main pathogen causing postharvest disease, which can cause black spot disease in dragon fruit (Castro et al., 2017), pears (Tian et al., 2006), peaches (Inoue and Nasu, 2000), and other fruits (Prusky et al., 1997; Prusky et al., 1999; Zhao and Liu, 2012) in the post-harvest preservation. This study found first that *A. oryzae* can cause infection on dragon fruit. Since *A. oryzae* is a ubiquitous environmental fungus, the infection observed in this study could be opportunistic. This research reported for the first time several pathogenic microorganisms of dragon fruit in Guizhou, indicating that the distribution of pathogenic microorganisms in dragon fruit varies with a geographical environment which on the other hand signifies the importance of the geography-specific plan for prevention and control measures.

*A. alternata* and *F. proliferatum* are two typical postharvest pathogenic microorganisms that can cause postharvest diseases in a variety of fruits (Konstantinou et al., 2011; Abd Murad et al., 2017; Castro et al., 2017; Wang et al., 2021), so we selected these two to test their susceptibility to the control agents, respectively. The results showed that *A. alternata* H4 exhibited the highest sensitivity to 430 g/L tebuconazole and the lowest sensitivity to 80% ethylicin, while *F. proliferatum* H8 showed the highest sensitivity to 45% prochloraz and the lowest sensitivity to *Bacillus cereus*. The results showed that 10% difenoconazole, 50% iprodione, 50% kresoxim-methyl, 80% ethylicin, and 430 g/L tebuconazole all had inhibitory effects on the two postharvest pathogenic microorganisms of pitaya to a certain degree. Natural antimicrobials obtained from plants can provide alternative materials instead of commonly used fungicides in a more sustainable and environment-friendly way (Romanazzi et al., 2012). This study demonstrated that the 10 edible and medicinal plant extracts all showed some inhibitory effects on the two representative pathogenic fungi, although they are much less potent than chemically synthesized fungicides. Chemical fungicides showing the most efficacy to tested pathogens can be good choices for field application; however, long-term single use of control agents will likely cause drug resistance of pathogenic microorganisms. Therefore, it is recommended to carry out field experiments using a combination of agents which would improve the control effect, but the study only conducted a sensitivity test indoors which may not represent the field effect.

Pathogenic microorganisms can infect fruits in many ways. For example, fungal spores can spread with the help of wind or insects to infect fruits. Therefore, cultivation and management should be strengthened at regular times, and attention should be paid to ventilation and light transmission to reduce the reproduction of fungi since the reproduction and accumulation of pathogenic microorganisms would cause more fruits to rot, aggravating microbial infections. At present, the postharvest preservation methods of dragon fruit mainly include low-temperature preservation, film preservation, chemical preservation, thermal treatments, and irradiation (Jalgaonkar et al., 2020). These preservation measures have problems such as being time-consuming and labor-intensive, high cost, chemical residues, environmental pollution, and food safety hazards. Plant extracts, especially those from edible or medicinal plants, with fungal inhibitory effects can find their use in postharvest disease prevention.

## Conclusion

The study isolated seven strains from diseased dragon fruits, and their ability to infect healthy dragon fruits was confirmed. Of them, *Penicillium spinulosum*, *Phoma herbarum*, *Nemania bipapillata* and *Aspergillus oryzae* were for the first time found to cause dragon fruit disease. In consideration of their prevalence in postharvest fruit diseases, *A. alternat*a H8 and *F. proliferatum* H4 were chosen as representative pathogens for the drug susceptibility test. Among the tested agro-agents and plant extracts, 430 g/L tebuconazole and 45% prochloraz were found to be the most potent fungicides against H8 and H4, respectively.

## Data Availability

The datasets presented in this study can be found in online repositories. The names of the repository/repositories and accession number(s) can be found in the article/supplementary material.
